# Brexit and Animal Welfare Impact Assessment: Analysis of the Opportunities Brexit Presents for Animal Protection in the UK, EU, and Internationally

**DOI:** 10.3390/ani9110877

**Published:** 2019-10-28

**Authors:** Steven P. McCulloch

**Affiliations:** Centre for Animal Welfare, University of Winchester, Winchester SO22 4NR, UK; Steven.McCulloch@winchester.ac.uk; Tel.: +44-(0)1962-827-465

**Keywords:** Agriculture Bill, animal welfare impact assessment, Brexit, Common Agricultural Policy, Conservative Party, fur farming, live animal exports, puppy smuggling, sentience policy, World Trade Organisation

## Abstract

**Simple Summary:**

The British people voted in 2016 to leave the European Union (EU). The UK has a unique history as a leader in animal protection policy. It has a relatively large economy and significant political power on a global basis. Brexit presents both threats and opportunities to animal protection in the United Kingdom (UK), EU, and internationally. This paper assesses the opportunities Brexit presents for animal protection in terms of five criteria. These are first, the political situation; second, regulatory changes; third, economic and trade factors; fourth, institutional considerations; and fifth, EU and international impacts. Brexit provides the opportunity to reform UK farming to promote high animal welfare outside of the EU Common Agricultural Policy (CAP). Brexit means the UK can ban live animal exports and the import and sale of fur products and foie gras outside of the EU. Leaving the EU permits the UK to have stricter requirements for the Pet Travel Scheme (PETS) to control puppy smuggling. Brexit provides an opportunity for the UK Government to reform policy-making for sentient animals. New sentience legislation could establish a fully independent UK Animal Welfare Advisory body and mandate Government to use animal welfare impact assessments on all policy that affects sentient species. Despite such opportunities, the UK Government appears uncommitted to major reforms. The drafting of the Agriculture Bill does not suggest a progressive animal welfare agenda. For live exports, the Government will consult on how to improve welfare, rather than outright prohibition. Similarly, rather than ban the import and sale of fur, the Government will use its influence to improve the welfare of fur-farmed animals outside the UK. Brexit provides some opportunities for animal protection. Pre-Brexit, the Government has not demonstrated the political will and commitment to realise these opportunities.

**Abstract:**

The British people voted in a 2016 referendum to leave the European Union (EU). Brexit presents threats and opportunities to animal protection in the United Kingdom (UK), the EU, and internationally. This paper discusses opportunities for animal protection in terms of five criteria. These are first, political context; second, regulatory changes; third, economic and trade factors; fourth, institutional- and capacity-related factors; and fifth, EU and international considerations. Brexit permits reform of UK agricultural policy outside of the Common Agricultural Policy (CAP) to reward high welfare as a public good. The Agriculture Bill, however, does not suggest a radical reform agenda for animal welfare. Brexit permits a ban on live exports, but the UK Government is consulting on improving welfare, not prohibition. Brexit provides an opportunity to ban the import and sale of fur, but the UK Government has signalled it will work to improve welfare in fur farming. Brexit permits the UK to prohibit the import and sale of foie gras, but the Government has stated a ban may be challenged at the World Trade Organisation (WTO). Brexit allows more stringent Pet Travel Scheme (PETS) requirements to reduce puppy smuggling. Lucy’s Law and stricter enforcement will also mitigate the problem. New sentience legislation provides the opportunity for a fully independent and properly constituted UK Animal Welfare Advisory body conducting animal welfare impact assessments and ethical appraisal. The Government has proposed sentience legislation but there is a major risk it will not be in place before the UK leaves the EU. The Government has expanded the remit of the Farm Animal Welfare Committee, which is not fully independent and is dominated by veterinary members and agricultural interests. Brexit provides some opportunities for animal protection with radical reform of agricultural policy, prohibition of live exports, and banning the import and sale of fur and foie gras. Pre-Brexit, the Government has not demonstrated the political will and commitment to realise these opportunities.

## 1. Introduction

The United Kingdom (UK) has been a member of the European Union (EU) since 1993 and, before that, the European Communities (EC) since 1973. UK and EU animal protection law and policy is highly integrated. The UK, as a powerful EU member state with a history of progressive animal protection regulation, has had substantial influence on the development of EU animal welfare law. Furthermore, around 80% of UK animal welfare laws are based on EU regulations, rules, and directives. Farming policy in the UK has been determined by the EU’s Common Agricultural Policy (CAP) since 1973. There is a substantial volume of trade in agri-products and live animals between the UK and EU. The UK’s departure from the EU, or Brexit, therefore has the potential for major impacts on animal protection in the UK, EU, and internationally.

The UK voted narrowly by 51.9% to 48.1% in a June 2016 referendum to leave the EU. David Cameron, who had campaigned to remain in the EU, resigned as Prime Minister of the UK immediately after the result. Theresa May replaced David Cameron as Prime Minister in July 2016. Theresa May’s Government gave notice of Article 50 to leave the EU in March 2017. May’s UK Government then negotiated the Withdrawal Agreement and Political Declaration with the EU. The EU signed a Withdrawal Agreement in October 2018. However, Theresa May was unable to pass her Withdrawal Agreement through Parliament. The EU granted Theresa May’s request for an extension of Article 50 until 31 October 2019. Without a withdrawal deal, the UK would crash out of the EU at the end of March 2019. Theresa May resigned as Prime Minister on 24 May 2019 based on her Government’s failure to ratify the Withdrawal Agreement in Parliament, poor UK local election results for the Conservative Party, and continued opposition within her Parliamentary party.

In July 2019, Boris Johnson was elected leader of the Conservative Party and appointed Prime Minister of the UK. Johnson has stated that the UK will leave the EU on 31 October with or without an exit deal with the EU [1]. Johnson appointed a right wing eurosceptic Cabinet to deliver Brexit. In late August, Johnson’s Government asked the Queen to prorogue, i.e., suspend, Parliament for five weeks prior to 31 October. The prorogation of Parliament at such a critical time in the UK’s history was highly controversial and those opposed accused the executive of silencing Parliament to force through a no-deal Brexit. John Bercow, the House of Commons Speaker, called the suspension of Parliament a ‘constitutional outrage’. The Government claimed the prorogation was for a Queen’s Speech on October 14 to set out a new legislative agenda [2].

On 4 September, Members of Parliament (MPs) took control of the House of Commons business agenda. Johnson removed the whip from 21 Conservative MPs, effectively expelling them from the party, leaving his Government without a majority in Parliament [3]. Parliament then passed the European Union (Withdrawal) (No. 2) Act, also known as the Benn Act, to prevent the UK leaving the EU without a deal on 31 October. The Prime Minister called for a general election, but on 5 September the House of Commons did not vote for the two thirds majority needed under the Fixed Term Parliament Act 2011. Opposition parties suspected Johnson would use the general election to force through a no-deal Brexit and did not vote for the motion [4].

The Government’s decision to prorogue Parliament was challenged in the English and Scottish High Courts. The High Court of England ruled that that the decision to prorogue Parliament was a political matter and non-justiciable. Scotland’s Court of Session, however, found that the decision was justiciable and found against the Government [5]. On 24 September 2019, the UK Supreme Court ruled that the Government had acted unlawfully in proroguing Parliament [6]. The Supreme Court found that the prorogation prevented Parliament from fulfilling its constitutional role to scrutinise the executive. MPs returned to Parliament on 25 September amid calls for Boris Johnson to resign as Prime Minister [7].

Parliament was prorogued again on 8 October ahead of the Queen’s Speech to be held on 14 October 2019. The Agriculture Bill 2017–19 [8], the Trade Bill 2017–19 [9], and the Animal Welfare (Sentencing) Bill 2017–19 [10] did not complete their passage through Parliament so fell with the end of the session. This is because Bills that have not completed their passage through Parliament are not carried over into a new session unless a carry-over order is agreed [11].

In the Queen’s Speech, the Government announced it would introduce an Agriculture Bill and a Trade Bill that appear to have the same policy objectives as the 2017–19 Bills. The Queen’s Speech also included a new Animal Welfare (Sentencing) Bill, again to increase maximum sentencing for animal cruelty from six months to five years [12]. The Government would also ensure animals are recognised as sentient beings and animal welfare is taken into account in relevant government policy making. The Queen’s Speech included a measure to consult on improving the welfare of transported live animals, a call for evidence on keeping primates as pets, and a consultation on banning the import and export of trophies from the hunting of endangered animals [13].

Boris Johnson agreed a draft withdrawal deal with the EU on 17 October 2019. The deal was similar to Theresa May’s Withdrawal Agreement [14]. However, the customs union backstop had been scrapped and Northern Ireland would be subject to a dual tariff regime with the EU and the UK [15]. On 19 October Parliament voted to withhold approval for the Government’s withdrawal deal. It meant that Boris Johnson was forced to write to the EU to request an extension of Article 50 until 31 January 2020 [16].

The Government published its Withdrawal Agreement Bill on 21 October 2019. The Bill passed its second reading in Parliament on 22 October. The Government also tabled a motion to pass all stages of the Bill through Parliament within just three days. Parliament voted against the Government’s timetable because there would be insufficient time to scrutinise the Bill [17]. This meant that the UK would not leave the EU on 31 October. The political situation, and therefore the potential impacts of Brexit on animal protection, remains unstable at the time of writing in October 2019.

Brexit presents both threats and opportunities to animal protection policy. This paper builds on discussion of the legal and political context of Brexit and animal protection [18], and an analysis of threats to animal protection in the UK, EU, and internationally [19]. Section 2 describes a framework to assess the impacts of Brexit on animal protection. Section 3, Section 4, Section 5, Section 6, Section 7 and Section 8 assess the opportunities that Brexit presents for animal protection. Section 9 concludes the discussion in the paper.

## 2. Framework

How Brexit will impact animal protection is highly complex and there are significant uncertainties. Brexit will affect farmed, research, companion, and wild categories of animals. The form of the UK’s departure from the EU and its future trading relationship with the EU and non-EU nations will have a major impact on animal protection. Based on the economic size and power of the UK and the EU, and the UK’s historical role as a global leader in animal welfare, Brexit will impact animal protection not only in the UK and EU, but also internationally. Ultimately, how Brexit impacts animal protection will be determined by the decisions of key political and other official actors in London, Brussels, and Washington. The following framework, reproduced from McCulloch [18], is used in this paper to assess the impact of Brexit on animal protection.

1. What is the current relationship between the UK and the EU in animal health and welfare policy, i.e., what is the status quo?
2. What is the political context of Brexit, i.e., what are the political considerations that are likely to determine the impact of Brexit on animal protection?
3. What are the threats and opportunities to animal protection of Brexit?
4. What are the threats and opportunities to animal protection of Brexit to different categories of animals?
5. What are the threats and opportunities to animal protection of different forms of Brexit?
6. What are the threats and opportunities to animal protection of Brexit geographically, i.e., in the UK, the EU, and internationally?
7. What are the magnitudes of the various threats and opportunities of Brexit?
8. How likely are the various threats and opportunities of Brexit to animal protection to materialise?
9. All things considered, will Brexit be a net positive or negative for animal protection in the UK, EU, and internationally?

Questions 1–5 of the framework were investigated in McCulloch [18]. Questions 6–8 of the framework, with respect to the threats that Brexit poses to animal protection, were investigated by McCulloch [19]. This paper investigates questions 6–8 of the framework, with respect to the opportunities that Brexit presents for animal protection.

## 3. Assessment of the Opportunities that Brexit Presents for Animal Welfare

Table 1 outlines the major opportunities that Brexit presents for animal protection. The key opportunities are categorised as political, regulatory, economic and trade, institutional- and capacity-related, and EU and international factors. The discussion of these opportunities in the text follows the same order as that presented in Table 1. The order is intended to be logical, beginning with the broader political context of Brexit, moving to regulatory changes, through to economic and trade considerations, and then institutional- and capacity-related factors. The final category in the table moves to the discussion of how Brexit might impact EU and international animal protection. These categories also mirror those in Table 1 of McCulloch [19] discussing the threats posed by Brexit to animal protection.

## 4. Political Factors

### 4.1. The UK Is a World Leader in Animal Protection

The UK has a unique history as a global leader in animal welfare. In Martin’s Act 1822, it passed some of the first animal protection laws. In the Royal Society for the Prevention of Cruelty to Animals (RSPCA), it established the first humane society to protect sentient creatures in 1824 [20]. In the 1960s, the Brambell Committee’s report on intensive farming paved the way for animal welfare science and official government advisory bodies [21]. The UK’s anti-cruelty and animal welfare laws, humane animal protection groups, and government advisory bodies have been replicated and emulated throughout the world. Early pioneers in animal welfare science worked at leading UK universities, including Cambridge, Oxford, Bristol and Edinburgh [22].

The UK has had a major positive impact on animal protection in the EU. It lobbied for animals to be recognised as sentient beings during its Presidency year. The UK influenced bans on veal crates (2007), barren battery cages (2012), and the regulation of sow stalls (2013). It is to a significant degree due to the UK’s influence that the EU now has the most progressive animal welfare laws in the world [18]. These laws have had a major impact on animals because of the large market of 510 million citizens and consumers across 28 member states. 

The EU generally sets minimum standards, and member states are able to make more progressive regulations based on these. The UK has, in many cases, taken this opportunity. For instance, Britain requires broilers to be given more space than is required by EU law. In some cases, the UK has introduced reforms much sooner than the EU. The UK prohibited veal crates in 1990 and sow stalls in 1999, for example. Furthermore, whereas the EU permits sow stalls to be used for four weeks, the UK has a complete ban.

Of course, membership of the EU has also acted as a constraint. EU membership means pooling sovereignty and not always achieving the UK’s preferred policy in all cases. Brexit therefore means the UK will be free to make laws that surpass the EU in areas where it was previously constrained. For instance, the UK will no longer be constrained by the EU if it wishes to ban live animal exports (Section 5.2), or the import and sale of fur products (Section 5.3) or foie gras (Section 5.4). Brexit means the UK will leave the EU Common Agricultural Policy (CAP) and can reform farming policy to promote animal welfare (see Section 5.1) [23].

It is important, however, to scrutinise how far the UK has been constrained by the EU and whether Brexit will lead to major reforms. In general, member states can mandate higher standards when implementing EU animal protection laws. The UK has implemented legislation earlier or mandated higher standards in a number of cases, as described above. However, it was claimed that the UK’s earlier implementation of the ban on sow stalls and tethers in 1999 resulted in importing cheaper pork and bacon from Denmark and other EU states, and harming the UK pig industry. For this reason, the UK has since resisted ‘gold-plating’ EU legislation in animal health and welfare to improve competitiveness [24,25]. 

In relation to this, after Brexit, the UK will continue to be a member of the WTO and must abide by its rules. Based on previous WTO case law, it is uncertain how the WTO would judge any UK restrictions on imports based on animal welfare standards. Hence, it is possible that after leaving the EU, the UK Government may continue to resist higher welfare standards, but this time not due to membership of the single market but because of WTO rules. The following sections discuss potential opportunities for improvements in animal protection, including possible barriers to reform.

### 4.2. Inherent Opportunities in Massive Political Change

Brexit has presented the opportunity for the UK to step back and take a comprehensive review of its animal protection policy [23]. Policy issues high on the agenda have included sentience legislation, agricultural policy, and live animal export. These issues are directly related to the UK’s relationship with the EU. As discussed in Section 5.1, EU membership has prevented the UK from having its own agricultural policy or from banning live exports. Furthermore, sentience policy has been high on the agenda because leaving the EU means the UK will no longer be bound by Article 13 of the Treaty of Lisbon. The latter presents a major threat in the form of the loss of the recognition of animal sentience and the duty of government to pay full regard to animal welfare [19]. It also presents an opportunity for the UK to reform sentience policy, such that it is more effective than Article 13 [26].

The focus on reforming sentience policy has led to substantial lobbying for the recognition of decapod crustaceans, such as crabs and lobsters, and cephalopods, such as octopuses and squid, as sentient animals [23,27]. EU membership does not prevent the UK recognising these creatures as sentient beings and formulating policy to protect them. Indeed, the UK Animal Welfare Act permits the minister to add species under Section 1 if they are satisfied that scientific evidence supports the claim that they are capable of experiencing pain and suffering. Despite an increasing body of evidence suggesting sentience in decapod crustaceans and cephalopods, government ministers have not added additional species to the list. Animal protection non-governmental organisations (NGOs) such as the RSPCA, World Animal Protection (WAP), and Crustacean Compassion have therefore lobbied for decapod crustaceans and cephalopods to be explicitly included in any new sentience legislation that is introduced.

### 4.3. Government Has Made Progressive Policy and Positive Policy Statements About Animal Welfare

Recent Conservative Governments have made some progressive policies on animal protection. These have included mandatory closed-circuit television (CCTV) in abattoirs, prohibiting microbead plastics in the UK, banning the sale of ivory, and passing a law to ban the third-party selling of puppies and kittens. Furthermore, the Government has made a number of positive statements specifically related to Brexit and animal protection. These policy statements generally commit to maintaining, and in some cases improving, animal protection post-Brexit. The former Department for Environment, Food and Rural Affairs (Defra) Secretary Michael Gove, for instance, made the following statement:

This Government is committed to the very highest standards of animal welfare. As the Prime Minister has set out, we will make the United Kingdom a world leader in the care and protection of animals… This government will continue to promote and enhance animal welfare, both now and after we have left the EU.

[28]

Policy statements should indicate government direction and can be referred to by lobby groups and the public to hold democratic governments to account. Policy statements can also be used, however, by governments to placate lobby groups and depoliticise controversial issues, because they are essentially non-binding. For these reasons, policy statements should be scrutinised in their broader context. 

Furthermore, governments and ministers can and do change; in 2019, Boris Johnson replaced Theresa May as Prime Minister and Theresa Villiers succeeded Michael Gove at Defra. Related to this, Boris Johnson’s Government is not bound by policy statements made in Theresa May’s 2017–19 Government. Politically, his government should respect the commitments in the Conservative Party’s 2017 manifesto, since he has not been given a fresh mandate in a general election. However, the Conservative Party 2017 manifesto only includes a broad statement to ‘continue to take action to improve animal welfare’ (p. 26) [29]. 

One of the major threats to animal welfare is the import of agri-products to the UK raised in lower welfare conditions in nations such as the United States (US) [19]. The Government had accepted an amendment to the Trade Bill 2017–19, which stipulated that imports must meet standards in existing free trade agreements [30]. The Trade Bill 2017–19, however, fell with the prorogation of Parliament on 8 October 2019.

In the 2019 Queen’s Speech to introduce the new Parliamentary session, the Government announced it would introduce a Trade Bill that appears to have the same policy objectives as the 2017–19 Bill. The background briefing to the Queen’s Speech states that the purpose of the Trade Bill is to protect UK businesses and consumers from unfair trade practices or surges in imports. It does not, however, specifically mention animal welfare as the 2017–19 amended Bill did. Furthermore, the Government has not yet passed sentience legislation that will confer important duties on ministers during post-Brexit trade negotiations.

## 5. Regulatory Changes

### 5.1. Reform of UK Agricultural Policy to Promote Animal Welfare

#### 5.1.1. Common Agricultural Policy

The EU CAP was established in 1962 by founding members of the European Community, and provides financial support to farmers [31]. Under Article 39 of the European Union Treaty, the objectives of the CAP are to increase agricultural productivity; to ensure a fair standard of living for farmers; to stabilise markets; to ensure food supplies; and to ensure affordable prices for consumers [32]. The CAP accounts for 38% of the total EU budget and the UK is a net contributor to the CAP [33]. Pillar 1 payments (the Basic Payment Scheme in England) are paid based on land or farm size. Pillar 2 payments aim to improve competitiveness of agriculture, ensure sustainable management of land, combat climate change, and support rural communities [34].

Despite major reforms, the CAP continues to be criticised for a number of reasons. First, payments based on land area owned means wealthy land owners are subsidised by the taxpaying citizen. Many wealthy landowners in the UK receive over £500,000 annually in CAP subsidies, and some even receive over £1 million [35]. Secondly, the CAP has been criticised as a protectionist policy that has prevented farmers in African and other developing countries to access EU markets [36]. Thirdly, the CAP has supported intensive farming practices and monoculture agriculture [37,38]. Critics argue that the CAP has promoted environmental degradation and wildlife destruction [39].

Further criticisms relate to having a single agricultural policy to cover 28 member states with different geography, climate, and economies, from the UK and Germany in the North to Greece and Spain in the South. In addition, the CAP became unwieldy and difficult, both for member states to administer and for farmers to negotiate. It is based on such criticisms of the CAP that the UK Government proposed a reformed agricultural policy for when it leaves the EU.

#### 5.1.2. Health and Harmony Command Paper

The UK Government announced its intention to introduce its Agriculture Bill in the 2017 Queen’s Speech. It then published its *Health and Harmony: the future for food, farming and the environment in a Green Brexit* white paper in February 2018. The paper lays out a vision for England’s agricultural policy for the following decade and how its Agriculture Bill would do this. At the same time, the Government issued a consultation on *Health and Harmony*. In the Foreword, Michael Gove writes that leaving the EU provides a ‘once-in-a-generation’ opportunity to reform British farming. The UK Government wants a ‘more dynamic, more self-reliant’ farming sector, it wants to increase exports and deliver a healthier environment (p. 6) [40].

‘Public money for public goods’ is the key principle of the Government’s vision. The public goods listed in Section 5 of *Health and Harmony* include improved soil health; improved water quality; better air quality; increased biodiversity; climate change mitigation; and enhanced beauty, heritage, and engagement with the natural environment. The document then states ‘In addition to environmental enhancement, our new policy *could* also work towards achieving any or all of the following outcomes’ (italics mine). The document then lists ‘Better animal and plant health and animal welfare’, including ‘World-class animal welfare’ and ‘High animal health standards’ [40] (pp. 33–34). Section 7 of *Health and Harmony*, ‘Fulfilling our responsibility to animals’, states:

*Animal welfare is one of the public goods we could support in the future. During the ‘agricultural transition’, we could pilot schemes that offer targeted payments to farmers who deliver higher welfare outcomes in sectors where animal welfare largely remains at the legislative minimum*.

(p. 43) [40]

Over two thirds (68%) of respondents thought there was a strong case for pilot and other schemes to incentivise animal welfare. In its submission, the RSPCA argued for subsidies to be paid for welfare above the UK regulatory requirement:

*The RSPCA believes the new support system should reward farmers with public money only if they go beyond current standard industry practice, i.e., neither rewarding producers for ‘business as usual’, nor for just being legally compliant. Current cross compliance already includes adherence to the Welfare of Farmed Animals (England) Regulations 2007*.

(p. 21) [38]

The consultation asks ‘Should government set further standards to ensure greater consistency and understanding of welfare information at the point of purchase?’. In response, 40% answered ‘yes, as long as it does not present an unreasonable burden to farmers’; 32% answered ‘yes’ categorically; and 14% answered ‘no, it should be up to retailers and consumers’. The RSPCA argues there is a ‘wealth of evidence’ that method of production labelling is both something that British consumers want and that drives higher welfare (p. 20) [38]. It uses the example of mandatory labelling of the method of production for eggs across the EU enabling consumer choice and driving growth in the cage-free industry, which has improved the wellbeing of millions of laying hens.

#### 5.1.3. The Agriculture Bill 2017–19

The Government introduced its Agriculture Bill to Parliament in September 2018:

The Agriculture Bill sets out how farmers and land managers will in future be paid for “public goods”, such as better air and water quality, improved soil health, higher animal welfare standards, public access to the countryside and measures to reduce flooding.

[41]

The Agriculture Bill is the first major piece of law guiding UK agricultural policy since the 1947 Agriculture Act was passed by Clement Atlee’s post-war Labour Government. The Act promoted agricultural productivity and provided assured market prices and adequate working conditions for British farmers. The Agriculture Act 1947 and later CAP succeeded in promoting agricultural productivity. Both the UK and later the European Communities and EU agricultural policy was successful based on the criterion of plentiful and affordable food [42].

Despite this, high agricultural productivity was associated with costs to the environment, human health, and animal welfare [43]. Such costs are ‘externalised’ as they are not accounted for in the cheaper monetary price of food. These externalised costs, including animal welfare, are public goods because they benefit society but do not provide a monetary profit. The former Farm Animal Welfare Council (FAWC) economist John McInerney has described how farm animal welfare is an externality, as well as a public good, in a 2004 government-commissioned report [44]. Animal protection NGOs, including the RSPCA [38] and Compassion in World Farming (CIWF) [45], as well as the British Veterinary Association (BVA) [46], have called on the Government to recognise animal welfare as a public good.

The Agriculture Bill does recognise animal welfare as a public good in Part 1. Specifically, the Bill states in Part 1 clause 1(1)(f) that the Secretary of State may give financial assistance for ‘protecting or improving the health or welfare of livestock’. The Government plans to replace the Direct Payment System, based on total land, with an Environmental Land Management system. The Bill provides powers for ministers to work with farmers to pilot new approaches to land management. The Government argues that the Bill lays the foundations for a ‘Green Brexit’ [41]. The Government guaranteed the Direct Payments of CAP in 2019 and 2020. There will then be a seven-year transition period from 2021 when payments are gradually phased out.

#### 5.1.4. Reception of the Agriculture Bill and Potential for Reform

The Labour Party opposed the Government’s Agriculture Bill in Parliament. Sue Hayman, the environment spokesperson, criticised the Bill for lacking detail, not guaranteeing continued funding post-2021, and for having no vision for future food policy [47]. At the Bill’s committee stage, the RSPCA was broadly supportive of the Bill. It welcomed the recognition of animal welfare as a public good and the inclusion of funded pilot schemes to incentivise animal welfare [48].

Despite this, the RSPCA had a number of concerns. First, the Bill does not contain a financial framework for support post-2022, meaning there is no guarantee to fund public goods such as animal welfare. Secondly, the Bill does not contain a provision to stop imports of products raised with lower welfare standards to the UK. Michael Gove stated in Parliament that future trade deals would not lower welfare standards: ‘We will not enter into trade or other agreements that undercut or undermine the high standards on which British agriculture’s reputation depends’ [47]. However, Gove’s statement appears to refer to UK agriculture and not the import of lower welfare meat, eggs, and dairy from other countries. The Government accepted an amendment to the Trade Bill 2017–19, which stipulated that imports must meet standards in existing free trade agreements. However, the Bill covered existing free trade agreements alone. Furthermore, the 2017–19 Trade Bill fell with the prorogation of the Parliamentary session. The 2019 Queen’s Speech included a new Trade Bill, but the briefing notes did not refer to animal welfare.

Benton et al. [49] have warned about the risk of the development of a post-Brexit two-tier system for food standards in the UK:

*A two-tier regulatory system could emerge whereby the UK produces food at higher standards but, under new trade relationships, imports cheaper and potentially lower-quality food from countries with reduced welfare or environmental standards*.

(p. 31) [49]

The RSPCA has recommended that the Government amends Clause 27 of the Agriculture Bill, such that improvements in farm standards in the UK do not leave UK farmers at a competitive disadvantage. The Parliamentary Environment, Food and Rural Affairs Committee (EFRA Comm) tabled an amendment to the Agriculture Bill to ensure parity of animal welfare in imports. The Government opposed amendments to the Agriculture Bill to ensure parity of standards.

A further concern about the Agriculture Bill relates to the stated objective of the Government to increase productivity and exports. The objective raises the concern of further intensification of agriculture and the use of techniques such as genetic modification and cloning. The former Defra secretary Michael Gove had been supportive of genetic modification and similar techniques [50]. Furthermore, Boris Johnson, in his first speech as Prime Minister, stated the following: ‘let’s start now to liberate the UK’s extraordinary bioscience sector from anti genetic modification rules and let’s develop the blight-resistant crops that will feed the world’ [51]. Leading animal protection groups are implacably opposed to genetic alteration techniques. For instance, the RSPCA claims that productivity is already pushing animals to their physical and metabolic limits, and that genetic alteration will lead to further suffering [38].

At the time of writing in October 2019, the Agriculture Bill fell at the prorogation of Parliament to end the session. The Queen’s Speech included an Agriculture Bill that appears to have the same objectives as the 2017–19 Bill. However, the background briefing document to the 2019 Queen’s Speech does not include reference to animal welfare in the section on the Agriculture Bill. The briefing discusses public goods but only in the context of environmental public goods. This is further indication that the Government does not plan to use the Agriculture Bill for a serious farm animal welfare reform agenda.

### 5.2. Prohibition of Live Animal Exports

The live export of farmed animals is a flagship animal protection issue in the UK. Animals are exported for slaughter, further rearing (or fattening), and breeding. Since the 1970s, British animal rights protestors have demonstrated against the export of live animals to Europe and beyond. Jill Phipps was 31 when she was crushed to death obstructing a lorry transporting live veal calves in 1995 [52]. Animal protection groups such as CIWF [53] and the RSPCA [54] have maintained high profile campaigns opposed to live animal transport. The BVA is also opposed to the practice, and argues that animals should be transported ‘on the hook, as meat, not the hoof, as live animals’ [55].

EU member states cannot prohibit live exports of animals, based on the freedom of movement of goods. Live animals, though recognised as sentient in EU law, are traded as commodities in the EU single market. The principle has been tested in UK courts. In 2012, in the UK port of Ramsgate, a lorry that was to transport sheep to Europe was deemed unsuitable at inspection. More than 500 sheep where held at Ramsgate port in temporary pens. During the incident, 45 sheep died; 43 were euthanised and two drowned after four fell into the water [56]. Thanet Council then prevented further export of live animals from Ramsgate. However, the UK High Court ordered Thanet Council to permit exporters to continue to use Ramsgate port.

#### 5.2.1. How Many Live Animals Does the UK Export?

Between 2000 and 2016, there was a substantial reduction in the number of live farmed animals exported from the UK. The RSPCA reports that the total number of farm animals exported from the UK to the EU for fattening or slaughter declined from 752,000 at the turn of the century to 43,000 in 2016. The majority of farmed animals exported from the UK are sheep, with a smaller number of cattle. The trade in calves declined from 83,000 in 2006 to 6000 in 2015. This is because it is cheaper to raise calves in the UK, and due to fears of spreading bovine TB in some EU countries [57].

Furthermore, large numbers of farm animals are transported across the UK/Irish border in both directions. For instance, 23,000 cattle were exported from Northern Ireland to the Republic of Ireland in 2017. Over half of these were originally from the Republic of Ireland. They had been born in the Republic of Ireland and exported to Northern Ireland for fattening, before returning back across the border again into the Republic to be slaughtered. The large volume of trade in live animals between the Republic of Ireland and Northern Ireland reveals how integrated agriculture is on the island of Ireland and the complexities it may cause in Brexit negotiations [57].

#### 5.2.2. EU and UK Regulation on Live Animal Transport

The transport of live animals across the EU is controlled by Council Regulation (EC) No 1/2005 on the protection of animals during transport and related operations. It is implemented in the UK by the Welfare of Animals (Transport) (England) Order 2006 and by parallel legislation in Scotland, Wales, and Northern Ireland [58]. A review of Council Regulation (EC) No 1/2005 published in 2012 found that the policy had improved welfare, though significant problems remained [59].

Given that EU membership prevents the UK from banning live exports, Brexit provides an opportunity to prohibit the practice. A full trade ban is supported by animal protection groups such as the RSPCA and CIWF. The RSPCA is concerned about three broad issues: Firstly, there is significant potential for animals to suffer during transportation [60]; secondly, enforcement of Council Regulation (EC) No 1/2005 is lacking in many countries; and thirdly, animals may be transported to EU countries with lower welfare standards than the UK. Scottish calves, for instance, are routed through Northern Ireland to be exported through the Republic of Ireland to Spain. The Welfare of Farmed Animals (Scotland) Regulations 2010 requires calves of all ages to be provided with bedding, whereas Spanish law only requires calves to have bedding for the first two weeks [57]. Furthermore, some British animals are re-exported outside of the EU to countries with far lower welfare laws:

The prospect of Scottish calves being re-exported from Spain to Turkey, the Middle East and North Africa is deeply disturbing. Many animal welfare organisations have investigated slaughter in this region for years. Slaughter methods are routinely inhumane and in breach of the international standards on the welfare of animals at slaughter of the World Organisation for Animal Health.

[61]

The following section discusses how the political situation has evolved with respect to live animal export policy since the EU referendum.

#### 5.2.3. Political Context and UK Government Position on Live Animal Export

George Eustice, the minister for agriculture, has outlined in Parliament how the UK’s relationship with the EU affects its policy on live animal exports:

While we are in the EU, it would be against free movement rules to place an ethical ban on the export of live animals, but once we leave the European Union, we will be free to do so, if that is the decision of the UK Government; there will be nothing to stand in our way.

[62]

The Conservative Party’s 2017 general election manifesto included the statement ‘as we leave the European Union, we can take early step to control the export of live farm animals for slaughter’ (p. 26) [29]. As a backbench Member of Parliament, Theresa Villiers introduced the Live Animals Exports (Prohibition) Bill in October 2017. Private Members bills very rarely proceed to pass through Parliament as an act of law and are primarily devices to raise the profile of an issue through debate [63]. The Bill was withdrawn in November 2017 after its second reading [64].

A UK Government and Parliament petition to end the export of live farm animals after Brexit reached over 100,752 signatures and secured a debate in Parliament on 26 February 2018 [65]. The UK Government launched a consultation on live animal transport in April 2018 [66]. It also asked the Farm Animal Welfare Committee to review existing standards and make recommendations to improve animal welfare during transport. The Labour Party has stated that it will prohibit the live export of animals for fattening and slaughter. The ban would include an exemption to transport breeding animals in high welfare conditions and for transport across the Northern Irish border [67].

In July 2018, Boris Johnson resigned his position as Foreign Secretary in Theresa May’s Government. Johnson resigned after the Chequer’s Agreement, including the ‘common rule book’ between the EU and UK that included agricultural goods. Johnson wrote in his resignation letter that the common rule book led to the UK having the ‘status of a colony’ of the EU [68]. Later, in November 2018, Johnson wrote of a ‘Brexit deal sell-out’ protecting the ‘barbaric trade of sending live animals abroad for slaughter’. In his full length article written for the British newspaper *The Sun*, Johnson argued that the backstop arrangement in Theresa May’s Withdrawal Agreement meant that the UK would not be able to ban live animal exports [69].

Despite Johnson’s earlier pronouncements, the 2019 Queen’s Speech did not include a Bill to prohibit live exports. The briefing notes to the Queen’s Speech included a commitment to issue a consultation on ‘improving the welfare of live animals’ transported for slaughter (p. 102) [13]. CIWF stated that the omission of a Bill on live exports was ‘extremely frustrating’, especially given the Government’s many earlier statements on the controversial issue [70].

#### 5.2.4. Political and Economic Barriers to Prohibiting Live Animal Exports

The minister for agriculture, George Eustice, is quoted above stating that leaving the EU permits the UK to ban live animal exports. However, Eustice goes on to state that ‘it is a little more complex than one might think’ [62]. Eustice refers to the UK exporting breeding pigs, as well as the export of sheep and cattle for fattening and slaughter. He also refers to some species of farmed animals coping with long distance travel better than others. Indeed, the leading animal protection groups do not lobby for the prohibition of all live exports. The export of live breeding animals is considered to be necessary for the farming industry.

The farming industry is broadly supportive of live animal exports [56]. Live animal export is not a devolved issue and Defra reserves responsibility for making policy for Scotland, Wales, and Northern Ireland. The Scottish Government is opposed to banning live exports [71]. Trade in live animals on the island of Ireland is highly integrated, with substantial trade in cattle, pigs, and sheep in both directions [61]. Furthermore, the UK would not be able to prevent trade between Northern Ireland and the Republic of Ireland based on the Belfast Good Friday Agreement.

Animal protection groups campaign for a ban on live export for fattening, as well as slaughter. If the UK Government prohibits export for slaughter alone, this may leave a loophole for animals to be transported for fattening when they are, in reality, being exported for slaughter [72]. However, a complete ban on fattening and slaughter is more restrictive and will face greater opposition from the farming industry.

A final barrier relates to the political and economic uncertainties around Brexit. A no-deal Brexit, in particular, means major risks, including the EU erecting barriers to the UK’s largest export market [73]. All forms of Brexit are forecast to cause a negative economic impact on the UK [74,75,76]. If the UK were to permit the import of agri-products from nations such as the US or Australia, farmers will already be faced with increased competition from cheaper products [77]. Prohibiting the live export of animals will likely have some detrimental impact on some sectors of the UK farming industry. The farming industry may claim that it should not face additional—and what it will argue as unnecessary—further pressures related to banning live exports.

#### 5.2.5. The WTO and Live Exports

The WTO is an intergovernmental organisation based in Geneva, Switzerland. Its purpose is to promote free trade based on global trading rules [78]. WTO members can challenge other members if they believe trade measures are unfairly protecting home industries. As a member of the EU, the UK has a number of trade bans relating to animals, which will continue to be in place when the UK leaves the EU. These include a 2007 ban on the import of dog and cat fur and a 2013 ban on cosmetics tested on animals. These prohibitions have not been challenged at the WTO [57].

There is concern that if the UK Government were to ban live animal exports, it would be challenged at the WTO. A challenge would likely be related to Article XI of the General Agreement on Tariffs and Trade (GATT), which states that members cannot impose bans or restrictions on imports and exports. Furthermore, if the UK were to ban live export to Europe but permit continued export to the Republic of Ireland, it may contravene GATT Article I, which states that members cannot treat nations differently in trade measures related to the same product [57].

There have been twenty trade bans relevant to animal protection challenged and assessed at the WTO, including the shrimp–turtle case and the EU seal case. The RSPCA claims that the ‘overarching trend’ is that the WTO is increasingly accepting trade restrictions related to societal values, including animal welfare. Furthermore, the Brexit Taskforce has highlighted that since live exports from the UK go to the EU, it would need to be the EU that challenged the UK Government at the WTO. Given that animal welfare is a value the EU ‘proudly defends on the global stage’ [61], arguably it is unlikely that the EU would challenge the UK.

#### 5.2.6. Prohibiting Live Exports and the Republic of Ireland/Northern Ireland Border Issue

The Brexit Taskforce, a coalition of leading animal protection NGOs, has investigated the impact of Theresa May’s earlier Withdrawal Agreement and a no-deal Brexit on the live trade in farmed animals across the UK and Ireland border [61]. Brexit will mean there is a land border between Northern Ireland in the UK and the Republic of Ireland in the EU. Any ban on live exports from the UK will need to include an exception for trade across the Irish border between Northern Ireland and Ireland. This is both to respect the Good Friday Agreement and to continue the highly integrated trade in live farmed animals and horses within the island of Ireland.

The Withdrawal Agreement negotiated by Theresa May aims to enable continued trade between Northern Ireland and the Republic of Ireland. However, a ban on live exports in this context may be problematic because the UK would need to justify differential measures for the Republic of Ireland and other WTO members. In contrast, a no-deal Brexit means there is less of a problem for the UK to prohibit live animal export. In this case, there would likely be inspections of goods, including live animals, at the UK/Ireland border. This would be inconsistent with the Good Friday Agreement and also cause animal welfare problems related to longer transport times for checks at the border and for travel to abattoirs that are further away [61].

### 5.3. Prohibition of the Import and Sale of Fur Products

Fur farming was banned in England and Wales under the Fur Farming (Prohibition) Act 2000 and under parallel legislation in Scotland and Northern Ireland in 2002. The bans were enacted after the UK Farm Animal Welfare Council (FAWC) refused in 1989 to issue guidelines for the welfare of mink and foxes kept for fur farming on the basis that they are essentially wild animals and unable to carry out normal behaviours in farming systems [79]. Despite the UK-wide ban on fur farming, it remains legal to import and sell fur products in the UK; £55.6 million of fur products was imported into the UK in 2016. The top five countries exporting fur to the UK were Italy, France, Poland, China, and Russia. Humane Society International UK (HSI UK) estimates that the UK imports the equivalent of around 2 million whole animal furs annually. The UK imports animal skins from 1.72 million mink, 110,000 fox, 90,000 racoon dog, and 80,000 wild trapped animals (coyote, lynx, beaver, otter) [80].

#### 5.3.1. The Welfare of Fur-Farmed Animals

The EU Scientific Committee on Animal Health and Animal Welfare (SCAHAW) investigated the welfare of fur-farmed species in 2001 [81]. It investigated welfare conditions of mink, polecat/ferret, red fox, arctic fox, raccoon dog, coypu, and chinchilla. The SCAHAW found that husbandry systems caused ‘serious problems’ for all species reared for fur. It further recommended that cages and management should be ‘greatly improved’ for mink and foxes, to improve their environments based on their complex biological needs (p. 186) [81]. Pickett and Harris [82] have reviewed the evidence on how farming impacts the welfare of the American mink (*Neovison vison*), the red fox (*Vulpes vulpes*), and the arctic fox (*Vulpes lagopus*). They conclude that farming systems used for mink and foxes do not satisfy any of the Five Freedoms and that it is ‘impossible’ to meet the needs of mink or foxes in commercial fur farming (p. 6) [82]. Pickett and Harris recommend a ban as the only viable option to solve the welfare problems inherent in fur farming.

#### 5.3.2. EU Regulation

The EU banned the import and sale of products containing dog and cat fur in 2007. In 2009, the EU prohibited the import and sale of seal products [23]. The EU ban on seal product imports was challenged by Canada and Norway at the WTO. In the *EC-Seal Products* case, the WTO Appellate Body found that the ban was necessary to protect public morals under the General Agreement on Tariffs and Trade [83]. The EU seal fur and skin ban thus sets an important precedent in WTO law to restrict trade based on public morality related to animal welfare. EU directive 98/58/EC applies animal welfare standards to farmed animal production, including species such as mink and fox farmed for fur. EU regulation 1099/2009 applies requirements to protect the welfare of fur animals at the time of killing. However, there is no species-specific EU legislation for fur-farmed animals.

#### 5.3.3. UK Political Context

The Parliamentary EFRA Committee launched an inquiry into the UK fur trade in February 2018. The inquiry was prompted by investigations into the sale of ‘fake faux fur’ from rabbits, fox, and chinchilla in the UK market by Humane Society International UK, Sky News, and the BBC [84]. The Parliamentary EFRA Committee recommended that Government hold a public consultation on whether to ban the import and sale of fur [85]. The Government provided the following response to the Parliamentary Committee:

While the UK is a member of the EU it is not possible to introduce restrictions relating to the fur trade which are inconsistent with the Treaty on the Functioning of the EU and which impair the free movement of goods within the EU single market. There will be an opportunity for government in the future, once we have left the EU and the nature of our future trading relationship has been established, to consider further steps such as a ban on fur imports or a ban on sales.

(p. 7) [86]

In October 2017 a UK Government and Parliament petition to ban the sale of animal fur in the UK was launched [87]. The petition received 109,553 signatures and was debated in Westminster Hall in June 2018. The Labour MP Daniel Zeichner introduced the debate by stating that the UK has banned fur farming but now effectively outsources fur production to countries with weak or no animal welfare laws [88]. Furthermore, Zeichner argued that a prohibition on the import and sale of fur would be permitted as a member of the EU. Articles 34 and 35 of the Treaty of Lisbon set out the principle of the free movement of goods in the single market. However, there is a similar clause in Article 36 of the Treaty of Lisbon to that in the WTO rules, which provides exceptions for restricting trade in certain circumstances. Zeichner goes on to quote the Article 36 clause:

The provisions of Articles 34 and 35 shall not preclude prohibitions or restrictions on imports, exports or goods in transit justified on grounds of public morality, public policy or public security; the protection of health and life of humans, animals or plants… Such prohibitions or restrictions shall not, however, constitute a means of arbitrary discrimination or a disguised restriction on trade between Member States.

[88]

In his response to the debate, the Defra minister George Eustice stated that to ban the import of fur as an EU member would require the consent of all member states, or the UK would need to cede the decision to the EU Commission. He went on to say that for ‘political reasons’, it would be unlikely the UK would be able to achieve a ban as a member of the EU.

In November 2018, Defra responded to the Government and Parliament petition:

Regarding the fur industry specifically, we are working at an international level to agree global animal welfare standards and phase out cruel and inhumane farming and trapping practices. We believe this is the best way to prevent animal cruelty and that this approach will lead to a much higher level of animal welfare standards.

[87]

HSI UK was critical of the Government response. The organisation claimed that the way to phase out cruel and inhumane farming and trapping practices is ‘only achievable through the phase out of the industry itself’. HSI UK argues that the UK Government should take a ‘strong, symbolic and meaningful stand’ by enacting a complete ban on the import and sale of fur in the UK [80].

### 5.4. Prohibition of the Import and Sale of Foie Gras

Foie gras is produced by force feeding, or ‘gavaging’, ducks and geese with maize. The process produces a pathologically abnormal fat liver, which produces the pâté de foie gras that some consider to be a culinary delicacy. Foie gras is not produced in the UK, and the RSPCA claims it would be illegal to produce under UK animal welfare laws [89]. The UK does, however, import 180–200 tonnes per year, with 98% of the duck foie gras being produced in France. In French law, foie gras is classified as a protected cultural and gastronomical heritage of France [90]. France produces around 83% of duck foie gras and 25% of goose foie gras on a global basis [91]. Other EU producers include Hungary, Bulgaria, Spain, and Belgium.

Force-feeding of ducks begins when the birds are 12 weeks old and continues for 12–15 days. During this time, around 80% of ducks are housed individually in small wire cages [89]. Force-feeding involves restraining the bird to insert a pipe into its oesophagus two to three times daily. The EU SCAHAW concluded in a 1998 review that force-feeding in commercial foie gras production is ‘detrimental to the welfare of birds’ (p. 65) [92]. The EU SCAHAW made a number of recommendations, including avoiding feeding processes that resulted in a liver with impaired function or caused substantial discomfort; abolishing the use of individual cages; and rearing the animals such that they can engage in normal behaviours.

Rochlitz and Broom [93] reviewed the evidence on the welfare of ducks during foie gras production in France. Their review found that mortality is 2–6% higher in ducks during the force-feeding period compared to those reared for meat. The authors report posture and gait abnormalities, wing lesions, and contact dermatitis, which is often ‘widespread and severe’. Research documents inflammation of the oesophagus and pathological liver changes. The small cages and lack of sufficient water prevent the birds from carrying out normal behaviours. Rochlitz and Broom conclude that force-feeding causes ‘very poor welfare’ in ducks and recommend against the practice [93].

EU membership prevents banning the import and sale of foie gras under the free movement of goods principle. Outside of the EU, the UK could ban the import and sale of foie gras, which would be compatible with WTO rules based on public morality [23]. In October 2016, a few months after the EU referendum vote, the agriculture minister George Eustice outlined the legal situation in Parliament:

The EU has introduced controls on the production of foie gras. These controls do not ban the sale of foie gras and while the UK is a member of the EU we are subject to EU Treaty obligations in relation to the free movement of goods. Foie gras is not produced in the UK and this Government has made its views very clear that the production of foie gras using force feeding (or ‘gavage’ as it’s known in France) gives rise to serious welfare concerns. If any production were to occur, the Animal and Plant Health Agency would be asked to investigate and advise on any contravention of UK animal welfare laws.

[94]

In February 2017, Michael Gove was reported to be considering a ban on the import and sale of foie gras after Brexit [95]. The Labour Party has included a commitment to ban the import of foie gras in its 50-point animal welfare plan [67]. The Conservative MP Henry Smith led a debate in Parliament on the issue in June 2018. Smith referred to the problem of banning the import of foie gras whilst in the EU:

Government’s view is that an attempt to impose a unilateral ban on the import or sale of foie gras while we are still an EU member could be legally challenged as contravening provisions of the treaty on the functioning of the European Union. This country could then be referred to the Court of Justice of the European Union and face multiple damage claims from importers, exporters and other foie gras traders.

[90]

In July 2018, Michael Gove was criticised for stating that France might oppose any attempts by the UK to ban the import and sale of foie gras in a post-Brexit trade deal with the EU. The RSPCA called Gove’s statement ‘disappointing’ and cited a survey that found 63% of the public in favour of a ban [96]. Furthermore, the Conservative Government opposed a Labour Party amendment to the Agriculture Bill to ban the import and sale of foie gras [97].

### 5.5. Puppy Smuggling

#### 5.5.1. EU Law Relating to the Movement of Pet Animals

The movement of pets in Europe is controlled by three pieces of legislation. Regulation No 576/2013 is concerned with the movement of dogs, cats, and ferrets for non-commercial purposes. The regulation is also known as the Pet Travel Scheme (PETS), which has three key requirements. Pets need to be microchipped, vaccinated against rabies, and have a valid pet passport. The Pet Travel Scheme was relaxed in 2012 to harmonise travel across the EU. Prior to 2012, the UK required dogs to be vaccinated after 12 weeks of age, followed by a positive rabies titre blood test. Dogs were then permitted to travel after a further six months. This meant that dogs were ten months of age before being permitted to travel. Relaxation of these rules under Regulation 576/2013 meant dogs did not require a blood test and could travel after being vaccinated [23].

Council Directive 92/65/EEC, also known as the Balai Directive, regulates the commercial movement of pets in the EU. In addition to the requirements of microchipping, rabies vaccination, and holding a valid passport, there are more stringent requirements. Animals must originate from a registered business or holding, importers must obtain a certificate issued by the Trade Control and Expert System (TRACES), and the animal must be examined by a veterinary surgeon within 48 h of travelling to ensure they are fit to travel. Furthermore, anybody travelling with more than five dogs must travel under the Balai Directive.

Regulation No 1/2005 controls the commercial movement of animals when transported more than 50 km. Cats and dogs younger than eight weeks cannot be transported without their mother. Animals must be fit to travel and they must not be transported in conditions that are likely to cause injury or suffering [23,98].

#### 5.5.2. Puppy Smuggling as a Growing Problem

The changes to the Pet Travel Scheme in 2012 have led to substantial increases in the number of dogs travelling into the UK under the scheme. Defra figures reveal an 82% increase in the number of dogs registered outside of Britain in the first year after the controls were relaxed. There have been further increases in subsequent years, from around 138,968 in 2012 to 275,876 in 2016 [23,98]. The growing figure is, to a significant degree, due to pet puppies being smuggled from the EU for the UK market. The Dog’s Trust carried out investigations into puppy smuggling in Europe between 2014 and 2018. Based on its report *Puppy smuggling, a tragedy ignored* [99], Simona Lipstaite of the EU Dog and Cat Alliance describes the problem:

Puppies are bred in large numbers, often in horrific conditions in Central and Eastern Europe by corrupt breeders who are continuing to exploit the demand for these desirable breeds in Great Britain. They are brought into the country illegally at a young age in order to appear ‘cuter’ to buyers, with desirable breeds such as Pugs, Dachshunds, English and French Bulldogs making up 82% of those intercepted at the border.

[100]

The Dog’s Trust investigation reports how unscrupulous vets from EU nations such as Poland, Hungary, Romania, and Lithuania sign false documents that enable puppies to be transported on 30 h journeys across Europe to the UK. Ireland is also a major source of puppies smuggled into the UK [101]. The Dog’s Trust makes a number of recommendations to reform policy to prevent puppy smuggling. These include reintroducing the requirement for dogs to have a rabies blood test and wait six months prior to entry into the UK; reducing the number of dogs permitted to travel under PETS from five to two; improving enforcement at UK borders, with a shift of responsibility for enforcement away from carriers to government agencies; creating a database to record pet microchip numbers when travelling; and increasing penalties for puppy smuggling [101].

#### 5.5.3. The Political Context

In November 2017, Michael Gove stated that leaving the EU provides the UK with new opportunities to control the illegal trade in puppies. Gove’s statement was made after the Conservative Government had been widely criticised for rejecting an amendment to the EU Withdrawal Bill to continue to recognise animals as sentient after leaving the EU [102]. In 2018, the Labour Party pledged to control puppy smuggling in its 50-point plan. The opposition party stated that it would reintroduce rabies testing prior to entry into the UK, increase post-rabies testing to three months, and introduce a microchip database and record scanned animals on entry [67].

In December 2018, the Government announced that it would ban the third party sale of puppies and kittens in England [103]. The Government introduced the legislation to Parliament in May 2019 and it will come in to effect from April 2020. The legislation is named after Lucy, a Cavalier King Charles Spaniel breeding dog, who suffered due to being kept in terrible conditions on a Welsh puppy farm. The Government stated that the new legislation will also ‘deter puppy smugglers who abuse the Pet Travel Scheme (PETS) by bringing underage puppies into the UK which are then sold on for financial gain’ [104].

The Conservative MP Nigel Huddleston introduced a Westminster Hall debate on puppy smuggling in April 2019 [105]. In his response, the Defra Parliamentary Under-Secretary of State David Rutley referred to the need for Government to have ‘zero tolerance’ for those abusing PETS to smuggle dogs [106]. He cited Defra’s work engaging with the international community, improving enforcement, more stringent regulations, and public education to tackle puppy smuggling. Related to Brexit, Rutley stated:

Coming back to the “B” [Brexit] word, which a few hon. Members have mentioned, we will be considering our future approach to regulation in the context of the negotiations on our future relationship with the EU.

[106]

In July 2019, the Parliamentary EFRA Committee launched an inquiry into puppy smuggling [107]. The following section discusses how the withdrawal negotiations between the EU and UK affect the UK Government’s options for regulating puppy smuggling.

#### 5.5.4. The Impact of Brexit on Puppy Smuggling

The Canine and Feline Sector Group (CFSG) [108] has assessed the impact of two Brexit scenarios on trade in cats and dogs over the Irish and English Channel borders. The CFSG assessed the impact first of a deal between the EU and UK with a ‘free trade area’ for animals checked at borders, and secondly of a no deal with WTO rules applying. In the event of a deal, arrangements would be similar and it would be ‘difficult’ for the UK to ban imports of puppies from the EU. This is because the UK would not prevent imports across the UK/Ireland border in Northern Ireland. Banning imports from the rest of the EU and non-EU nations would thus breach WTO rules. However, the Political Agreement that has been negotiated between the EU and UK permits the UK to raise sanitary and phytosanitary (SPS) standards for animal health. Hence, it is possible for the UK to raise the minimum age at which dogs can be brought into the UK, together with resuming with a mandatory period of time after the rabies vaccination prior to import [108].

In the event of a no deal, the EU plans to treat the UK as an unlisted third country and apply regulations and tariffs. These include checks and controls for sanitary and phytosanitary standards. This would mean increased journey times, which may have a positive impact on reducing puppy smuggling in the short term. In a no-deal situation, the UK could ban the import of puppies, but under WTO rules, it would need to extend this ban to the Republic of Ireland to be compliant [108].

## 6. Economic and Trade Factors

The UK is a relatively large market, with 66 million consumers that are wealthy on a global basis. The EU and non-EU nations therefore have an economic incentive to export goods and services to the UK, just as the UK has the same incentive to export its goods overseas. In this respect, if the UK Government makes a firm commitment to animal welfare and insists on parity of standards with trade partners, it may result in animal welfare reforms as a result of future trade deals. Furthermore, the UK can use soft power to project its progressive animal welfare values on a more global basis.

The Government accepted an amendment in the Trade Bill 2017–19 to stipulate that imports meet the welfare standards of the UK. However, this applied only to existing free trade agreements. Furthermore, the Trade Bill 2017–19 fell with the prorogation of the Parliamentary session. The background briefing notes to the Queen’s Speech do not reference animal welfare in the section on the new Trade Bill. Furthermore, despite a further proposal to recognise animal sentience, there is a major risk that the UK will leave the EU without sentience legislation in place. If the UK leaves on no-deal terms, it will revert to WTO trading rules, which means it is far less likely that imports would be required to meet UK animal welfare standards.

## 7. Institutional- and Capacity-Related Factors

### 7.1. Sentience Legislation

The development of Brexit policy has shone a light on UK policy making with respect to sentience legislation. As discussed in McCulloch [18,19], the UK Parliament nationalised EU animal welfare laws in the EU Withdrawal Act. However, sentience policy, in Article 13 of the Treaty of Lisbon, was not carried over. Article 13 states that animals are sentient beings and mandates that member states pay full regard to animal welfare [109]. After initially claiming that there was no need for sentience legislation outside of the EU, the UK Government published its draft Animal Welfare (Sentencing and Recognition of Sentience) Bill in 2018. The Bill, however, was criticised by the Parliamentary EFRA Committee and subsequently withdrawn. These events have prompted animal protection organisations to focus attention to improve upon Article 13 and have a meaningful implementation process in place.

### 7.2. Animal Welfare Impact Assessment

If the Government is to pay regard to animal welfare, it should have a robust and systematic process in order to do this. Governments ordinarily use impact assessments to assess the impacts of policy options, for instance, economic impact assessment, environmental impact assessment, and social impact assessment. However, the UK Government has no formal and systematic process to assess the impacts of policy options on sentient species, which is entailed by the Treaty of Lisbon or equivalent sentience legislation. McCulloch and Reiss [110,111] have proposed mandatory animal welfare impact assessment (AWIA) for all public policy that significantly affects sentient species. Animal welfare impact assessments should be conducted for each species significantly affected for each policy option under consideration. The AWIA then feeds into the policy process so that decision-makers can properly account for how sentient species are affected by policy options.

### 7.3. Ethical Appraisal of Policy Options

Article 13 of the Treaty of Lisbon states that EU member states, and therefore their respective governments, must pay full regard to animal welfare. Governments, however, also have duties to pay regard to other parties, such as human society and the biotic environment. Since governments have a duty to pay regard to various groups or entities that legally deserve consideration, policy makers require a means of deciding on policy that is justifiable in terms of the distribution of positive and negative impacts. For instance, Clause 1.1 of the Government’s Animal Welfare (Sentencing and Recognition of Sentience) Bill states that Ministers of the Crown must have regard to the welfare needs of sentient animals. Clause 1.2 states that in discharging the duty Ministers must also have regard to the public interest. Hence, the Government necessarily requires both a body and a process whereby it can conduct or be advised on how to weigh these interests.

### 7.4. Reform of Existing Advisory Bodies

McCulloch and Reiss [112] have argued that animal welfare impact assessments should be conducted within Government. However, the Government should be advised by an independent Ethics Council for Animal Policy to inform the moral dimension of policy making that impacts sentient species. McCulloch and Reiss [112] have reviewed the existing animal health and welfare advisory landscape in the UK. We argue that existing bodies are not suitable for the ethical appraisal of policy options. For instance, the Animal Health and Welfare Board of England (AHWBE) is dominated by veterinary members and industry interests. The Farm Animal Welfare Committee (FAWC) has been brought into Defra as an expert committee and is no longer an arms-length independent Council. There is no advisory public body to inform the UK Government on the impacts on sentient wildlife species and no existing bodies have sufficient expertise in ethics, law, and related disciplines to carry out robust ethical appraisal.

McCulloch and Reiss have argued that it is the inherent moral dimension of animal health and welfare policy making that impacts sentient species, and a lack of ethical appraisal, that contributes to the ongoing highly controversial nature of public policy that impacts animals (e.g., in the UK Salmonellosis in eggs, Bovine Spongiform Encephalopathy (BSE), foot and mouth disease, bovine tuberculosis and badger culling). Such an independent Ethics Council for Animal Policy would be similar in concept to the highly respected UK Nuffield Council for Bioethics [112]. It should use established moral frameworks to provide Government with ethical appraisal of policy options [113,114,115]. The UK Government should review the animal health and welfare advisory body landscape during the process of leaving the EU.

### 7.5. Animal Welfare Advisory Body

A coalition of leading UK-based animal protection organisations, including the RSPCA, CIWF, and WAP, have called for a ‘Better deal for animals’. A UK Government and Parliament petition to recognise animal sentience and require that animal welfare has full regard in law received 103,866 signatures [116]. In the petition, the group called for a new Animal Welfare Advisory Council (AWAC) to provide advice to the UK Government and devolved administrations. The Council could do this through animal welfare impact assessments and ethical appraisal of policy options. The petition was to be debated in Parliament on 9 September 2019. However, the debate was cancelled as a result of the Government proroguing Parliament.

On 1 October 2019, the Government announced that the remit of FAWC was to be expanded to provide research and advice on pets and kept wild animals. FAWC has been renamed the Animal Welfare Committee (AWC) to recognise this change [117]. It is unclear whether the change was prompted by the ‘Better deal for animals’ campaign. If so, the AWC does not meet the requirement of independence from Defra. Furthermore, the expanded remit of the AWC, with the inclusion of a focus on pets and kept animals, will necessarily dilute the focus on farmed animals. This is a serious problem; in the UK, around 1 billion land farmed animals are raised and slaughtered each year [18]. In contrast, there are only 9 million dogs and 8 million cats kept as pets in the UK. The number of kept non-domestic pets is around 12 million [118]. It follows from the far larger number of farmed animals, the purposes that we use them for, and the conditions that they are kept in that farmed animals are better served by a dedicated public body to advise the Government on animal welfare. Such a public body must, however, be fully independent from Government and properly constituted. FAWC was not fully independent as a committee within Defra and was dominated by veterinary members and agricultural interests.

## 8. EU and International Factors

### UK Animal Protection Reform Promotes Animal Welfare at EU and International Levels

Post-Brexit, can the UK—as a more independent nation—have a greater opportunity to enhance animal protection in the EU and worldwide? First, the UK will need to maintain its current standards of animal protection. If the Government were to permit the import of lower welfare agri-products, for instance from the US, it is difficult to see how Brexit would lead to a net positive impact on animal welfare in the EU and internationally.

Secondly, it will be in negotiating trade deals that the UK can have the most impact on animal welfare. Given that the EU has, on a global basis, very high animal welfare standards, there is arguably more potential for the UK to influence animal protection positively in non-EU nations. Indeed, the Wildlife and Countryside Link and A-Law make the case that post-Brexit, the UK may have more influence if it continues to align itself with EU policy:

The UK and the EU will find they have in common their respective citizens’ desire to advance animal welfare; and the UK’s power to influence animal welfare standards worldwide will be so much greater if it seeks to do so in partnership, where appropriate, with the world’s biggest consumer market.

(p. 6) [23]

Hence, despite Brexit meaning the UK leaving the EU, the UK should still work in concert with the EU to have an impact on animal welfare at the international level. This follows from the market size, and therefore economic and political weight, of the UK and the EU. The UK population, at around 66 million, is considerable. However, the EU population is around 510 million, and the US population around 325 million. Given these disparities, the UK should continue to align itself with the EU to have an impact on animal protection at the international level.

Finally, there is potential for more specific UK-based reforms to have an impact at the EU or international level. Successful reform of UK agricultural policy to support high welfare may be emulated in other countries. If the UK prohibits live exports, or the import and sale of fur and foie gras, other nations may follow. Ministers often ask civil servants and experts what other nations do when they consult both informally and formally. For instance, one of the questions in the Animal Welfare (Sentencing and Recognition of Sentience) Bill asked how other states recognised animal sentience. Elected decision-makers generally prefer to follow tried and tested solutions to policy problems, rather than carve out entirely new solutions [119]. The prohibition in the UK of live animal transport, for instance, could therefore exert pressure on the EU or Australia to reform or prohibit the trade. A progressive animal welfare agenda in the UK would therefore have a far more substantial impact if replicated at the EU and international level.

## 9. Conclusions

Brexit is a major political upheaval for the UK, with threats and opportunities to animal protection. Given the unique history of the UK in animal protection and its relative economic size and political power, impacts will not be restricted to the UK, but will reach the EU and international level. This paper has assessed the opportunities that Brexit presents for animal protection. The paper assessed opportunities in terms of five criteria: First, political context; second, regulatory changes; third, economic and trade factors; fourth, institutional- and capacity-related factors; and fifth, EU and international considerations.

The Conservative Governments delivering Brexit have made some progressive animal protection policies and some positive policy statements about maintaining and improving animal welfare after the UK’s withdrawal from the EU. The major opportunities Brexit presents for animal protection are as follows: Reform of UK agricultural policy outside of the CAP to reward high welfare; banning live exports; prohibition of the import and sale of fur products; prohibition of the import and sale of foie gras; more stringent regulation of PETS to reduce puppy smuggling; and passing sentience legislation to give powers for a fully independent and properly constituted UK Animal Welfare Council to advise Government through animal welfare impact assessments and ethical appraisal of policy options.

The Government’s positions in these policy areas are as follows: The Agriculture Bill 2017–19 would give powers to ministers to support animal welfare as a public good. However, it is difficult to interpret the Bill as an agenda for radical animal welfare reform, and there are threats such as no commitment to funding post-2027 and the objective to increase productivity, which is generally associated with welfare problems. Furthermore, the briefing to the 2019 Queen’s Speech proposing a new Agriculture Bill does not reference animal welfare as a public good.

The Government has previously stated that all options are on the table for live exports. However, there are substantial legal and political barriers to a full ban and Government has signalled further control of the trade, rather than an outright ban. There was no Bill to ban live exports in the 2019 Queen’s Speech and the background briefing document stated that Government planned to consult on improving the welfare of exported live animals. The Labour Party has pledged to ban the live export of animals but maintain trade across the Irish border.

The Government has indicated it will look to use its influence on the international stage to reform fur farming, and not prohibit the import and sale of fur in the UK. This is disappointing, because the UK has a specific legal ban on fur farming and there is overwhelming and consistent public opposition to the UK fur trade. The scientific consensus is that fur farms cannot meet the welfare needs of species such as mink and foxes, which are not domesticated and remain essentially wild. The Labour Party has pledged to ban all fur in the UK. On foie gras, the Government has no policy position and has stated it will look at the issue after the UK’s departure from the EU. The Labour Party has pledged to ban imports of foie gras to the UK.

The Government has introduced Lucy’s Law to ban the third party sale of puppies and kittens in the UK. It has stated that it will review the PETS requirements after Brexit. The Government published the Animal Welfare (Sentencing and Recognition of Sentience) Bill in 2017. The Bill was later withdrawn after criticism from the Parliamentary EFRA Committee. The 2019 Queen’s Speech briefing includes a commitment to recognise animals as sentient and impose a duty on the Government to have all due regard to animal welfare in formulating and implementing policy. However, there remains a major risk that the UK will leave the EU without this crucial legislation in place in the post-Brexit period. This risks importing lower welfare agri-products from nations such as the US.

Animal protection groups have lobbied for a fully independent and properly constituted Animal Welfare Advisory body to implement sentience policy post-Brexit. Such a body could conduct mandatory animal welfare impact assessments and ethical appraisal of policy options. However, the Government has expanded the remit of FAWC (now the AWC) to provide advice on companion and kept wild animals. As a committee within Defra, FAWC/AWC is not fully independent and is dominated by veterinary members and agricultural interests. Furthermore, rather than use prospective animal welfare impact assessments the Government has indicated it prefers a retrospective ministerial report mechanism to discharge duties related to sentience. The Labour Party has pledged to strengthen animal welfare in UK law by appointing an Animal Welfare Commissioner to ensure that animal welfare is considered in policy making. It has also stated that it will recognise decapod crustaceans (e.g., lobsters) and cephalopods (e.g., octopuses and squid) as sentient animals.

Brexit provides some significant opportunities to reform animal welfare in the UK. The greatest positive impact would be if progressive reforms in the UK were to be replicated, or otherwise have a positive impact on the far larger numbers of sentient animals in the EU and internationally. However, the UK Government will need to commit to a progressive animal welfare agenda in order for positive impacts to materialise. The development of policy in the pre-Brexit period suggests the UK Government is not sufficiently committed for the major animal welfare reforms to be realised.

## Figures and Tables

**Table 1:** Summary of opportunities for animal protection presented by Brexit.

Factors	Opportunity	Notes
Political	The United Kingdom (UK) is a world leader in animal protection	The UK has a history of progressive animal protection and continues to be a world leader in animal welfare. The UK is constrained by European Union (EU) membership to improve welfare in some areas. Brexit means the UK can improve animal protection unilaterally.
	Inherent opportunities in massive political change	The political upheaval of Brexit permits comprehensive rethinking about agricultural and animal protection policy.
	Government has made progressive policy and positive statements about animal welfare	Progressive regulation, e.g., mandatory closed-circuit television (CCTV) in abattoirs. Public consultations, e.g., *Health and Harmony* on the future of food and farming. Policy statements on maintaining and improving animal welfare post-Brexit.
Regulatory changes	Agriculture Bill	After Brexit, the UK will be outside of the Common Agricultural Policy (CAP) and can subsidise animal welfare as a public good. This provides the opportunity to reform UK agricultural policy to improve animal protection.
	Prohibition of live animal exports	The UK is unable to ban live animal exports due to the free movement of goods principle in the EU single market. Brexit permits prohibition outside of the EU. Barriers include a challenge under World Trade Organisation (WTO) rules; industry and political opposition; loopholes, e.g., related to export for further fattening; and continued trade across the Northern Irish border.
	Prohibition of import and sale of fur products	Fur farming is banned in England and Wales under the Fur Farming (Prohibition) Act 2000 and across the UK under parallel legislation. Prohibition of import and sale of fur products may be possible in the EU under Article 36 clause of the Treaty of Lisbon. The Government has argued a ban as an EU member is unlikely and Brexit provides opportunity to consider prohibition. The Government, however, seems reluctant to ban and favours reformist agenda using international influence.
	Prohibition of import and sale of foie gras	The UK is unable to ban import and sale of foie gras due to the free movement of goods in the EU single market. Brexit permits prohibition outside of the EU. There is a relatively small market for pâté de foie gras in the UK. A ban may lead to a WTO challenge by large EU producers, e.g., France. Possible opposition to ban on libertarian grounds.
	Puppy smuggling regulation	Puppies are smuggled illegally from central and eastern EU states to the UK. Most puppies are smuggled under the non-commercial Pet Travel Scheme (PETS). Lucy’s Law, banning third party sale of puppies and kittens, will mitigate the smuggling problem. Brexit presents an opportunity for more stringent regulation and enforcement related to PETS.
Economic and trade	UK trade policy promotes high welfare	Post-Brexit, the UK Government insists on parity of animal welfare standards with trade partners. The UK market promotes improved animal protection in the EU and internationally.
Institutional- and capacity-related	Establishment of UK Animal Welfare Advisory body and mandatory animal welfare impact assessment	An independent body composed of members with significant expertise in animal welfare, ethics, and policy to inform government and the public. The body is related to sentience legislation and should have powers to hold the Government to account. A mandatory animal welfare impact assessment for all policy that significantly affects sentient species. Impact assessments are conducted for each species affected for all policy options under consideration.
	Reform of current expert advisory bodies	The Animal Health and Welfare Board of England (AHWBE) has insufficient expertise in animal welfare, ethics, and law for its role to provide strategic animal welfare advice to the Government. The Farm Animal Welfare Committee (FAWC)/Animal Welfare Committee (AWC) is not an arms-length body with genuine independence from the Government. The AHWBE and FAWC/AWC are dominated by veterinary members and agricultural interests. There are no public bodies to advise the Government specifically on the welfare of companion and wild animals.
EU and international	UK reforms in animal protection have positive influence on EU	A UK with a reformed agricultural support system rewarding high welfare, prohibition on live animal exports, and a ban on the import and sale of fur and foie gras is replicated at the EU level.
	UK reforms in animal protection have positive influence at international level	A UK with a reformed agricultural support system rewarding high welfare, prohibition on live animal exports, and a ban on the import and sale of fur and foie gras is replicated at the international level.
